# Exploring the Utility of the Muse Headset for Capturing the N400: Dependability and Single-Trial Analysis

**DOI:** 10.3390/s24247961

**Published:** 2024-12-13

**Authors:** Hannah Begue Hayes, Cyrille Magne

**Affiliations:** Psychology Department, Middle Tennessee State University, Murfreesboro, TN 37132, USA; hb3v@mtmail.mtsu.edu

**Keywords:** ERPs, N400, semantic processing, mobile EEG, reliability

## Abstract

Consumer-grade EEG devices, such as the InteraXon Muse 2 headband, present a promising opportunity to enhance the accessibility and inclusivity of neuroscience research. However, their effectiveness in capturing language-related ERP components, such as the N400, remains underexplored. This study thus aimed to investigate the feasibility of using the Muse 2 to measure the N400 effect in a semantic relatedness judgment task. Thirty-seven participants evaluated the semantic relatedness of word pairs while their EEG was recorded using the Muse 2. Single-trial ERPs were analyzed using robust Yuen *t*-tests and hierarchical linear modeling (HLM) to assess the N400 difference between semantically related and unrelated target words. ERP analyses indicated a significantly larger N400 effect in response to unrelated word pairs over the right frontal electrode. Additionally, dependability estimates suggested acceptable internal consistency for the N400 data. Overall, these findings illustrate the capability of the Muse 2 to reliably measure the N400 effect, reinforcing its potential as a valuable tool for language research. This study highlights the potential of affordable, wearable EEG technology to expand access to brain research by offering an affordable and portable way to study language and cognition in diverse populations and settings.

## 1. Introduction

Traditional research-grade electroencephalography (EEG) systems are known for their high temporal resolution, reliability, and multi-channel capabilities. Despite their valuable contributions to neuroscience research, these EEG systems present limitations that hinder broader application and inclusivity [1]. For instance, the relatively high upfront costs (from around USD 10,000 to over USD 80,000 for a new research-grade EEG setup) and the need for specialized training restrict access to academic institutions with limited resources. Furthermore, reliance on laboratory-based data collection limits the ecological validity of research findings and poses logistical challenges for participants. The time commitment involved in research participation and the inconvenience of traveling to a research facility can create barriers for potential participants, particularly those from marginalized communities who may face challenges accessing transportation to the research facilities or balancing research participation with other responsibilities. These limitations thus highlight the need for EEG systems that are both more accessible and inclusive, enabling real-world data collection from a broader range of participants, especially with the growing interest in utilizing EEG for diverse applications like brain–computer interfaces, neurofeedback, and mobile health monitoring.

The emergence of consumer-grade EEG devices, such as the InteraXon Muse 2, a four-channel EEG headband designed for neurofeedback during mindfulness exercises, has opened new possibilities for brainwave monitoring research. These affordable and portable devices have the potential to democratize research by enabling larger-scale studies, reaching diverse populations, and facilitating data collection in real-world settings [1]. However, using consumer-grade EEG for event-related potential (ERP) research, a specialized EEG analysis technique that examines neural responses time-locked to specific stimuli [2], presents unique challenges. Accurately capturing and interpreting these responses with low-cost, non-standard research-grade equipment requires careful consideration. Synchronization of experimental events with the continuous EEG data stream is crucial for ERP analysis, and low-cost systems may lack robust mechanisms for precise marking of stimulus onset. Additionally, ensuring sufficient data quality, including sampling rates and minimizing noise and artifacts, which can be amplified due to less precise electrode placement and fewer channels, is essential for the reliable identification of ERP components.

Despite these challenges, the InteraXon Muse 2 has gained traction within the scientific community due to several noteworthy factors. One key advantage is its compatibility with the Lab Streaming Layer (LSL) protocol [3,4], an open-source system allowing for precise synchronization of EEG data with stimulus presentation software like PsychoPy [5] and other Python-based packages, a crucial step in ERP research. Importantly, the Muse 2 provides open access to raw EEG data without requiring extra costs, unlike some other consumer-grade EEG systems that use proprietary formats and charge for access to raw data. This open access approach allows for data analysis with a broader range of software, including tools commonly used for research-grade EEG systems, ensuring compatibility, rigor, and reproducibility. In addition to these advantages, the Muse headset has been successfully implemented in various experimental paradigms for studying mindfulness, emotion classification, and substance use [6,7,8,9,10,11,12]. Studies have demonstrated promising EEG signal quality, including successful measurements of large ERP components like the N200 and P300 [13,14], even when directly compared to research-grade EEG systems [13]. However, the device’s limited electrode configuration raises concerns about its sensitivity to capture language-related ERP components [15], which are typically smaller in amplitude and more susceptible to variations in linguistic stimuli and individual differences in language processing [16].

This study aimed to address this gap in the literature by examining the efficacy of the Muse 2 for measuring the N400 component, a key ERP marker of semantic processing and the most widely studied component in language research [17]. The N400 is a negativity occurring between 250 and 600 ms post-stimulus onset, peaking around 400 ms. It is an integral part of the brain’s typical response to words and other meaningful stimuli, including pictures, faces, and environmental sounds [17]. In linguistic contexts, the N400 amplitude associated with words exhibits sensitivity to the degree of expectation. Specifically, its amplitude is larger when words are less anticipated and smaller when words are more predictable [17]. Researchers employ a variety of experimental paradigms and tasks to elicit the N400, with the priming paradigm being particularly common. For instance, the semantic relatedness judgment task (SRJT) employs a priming paradigm where pairs of words are presented in succession, the first termed the ‘prime’ and the second the ‘target’. Participants are then asked to assess how closely related the two words are in meaning [18,19,20]. Target words elicit larger N400 responses when preceded by an unrelated prime (e.g., “icing–bike”) compared to a related one (e.g., “pedal–bike”). Thus, the N400 offers valuable insights into how the brain constructs meaning from language, highlighting its critical role in integrating information and forming expectations. Additionally, the N400 shows promise for clinical applications as a biomarker for a range of learning disabilities (e.g., dyslexia [21]) as well as neurological and psychiatric disorders (e.g., Alzheimer’s disease [22], schizophrenia [23]).

Noteworthy for the present study, the N400 is measured relative to reference electrodes placed on the mastoids (i.e., part of the temporal bone behind each ear). Its broad distribution across the scalp suggests the potential for capture with various electrode configurations. While denser arrays may offer a more comprehensive measurement, a more limited selection of electrodes, such as those in the Muse headset, could thus prove effective. To assess the Muse’s capability for capturing the N400 effect, we used the SRJT because it is a well-established and reliable paradigm. We hypothesized that if the Muse is sufficiently sensitive, the N400 elicited by target words would be larger in unrelated word pairs compared to that in related word pairs.

## 2. Materials and Methods

### 2.1. Participants

Thirty-seven first-year college students participated in the study for course credits (see Table 1 for demographic information). All participants had normal or corrected-to-normal vision and no reported hearing deficits. This study was approved by the MTSU Institutional Review Board, and written consent was obtained from each participant.

### 2.2. Stimuli

The task consisted of 112 trials, during which participants viewed 56 related and 56 unrelated word pairs presented in random order. All word pairs used in this experiment were adapted from a previous SRJT study [23]. Lexical frequency for each word was quantified using log-transformed HAL frequencies obtained from the English Lexicon Project website, which provides norms derived from the HAL corpus [24]. The log HAL frequency for prime words was 8.70 (SD = 1.94), and for the target words was 10.50 (SD = 1.28). To assess the semantic relatedness between prime and target words, we used the Gensim python library [25] to access the pre-trained word embedding model “fasttext-wiki-news-subwords-300” [26] and compute cosine similarity. This model represents words as dense vectors in a 300-dimensional space, capturing semantic relationships based on their co-occurrence patterns in a large text dataset. Cosine similarity, computed between the vector representations of each word pair, ranges from 0 to 1, with higher values indicating greater similarity. The mean cosine similarity was 0.67 (SD = 0.11) for related word pairs and 0.30 (SD = 0.08) for unrelated word pairs.

### 2.3. Experimental Procedure

Participants were seated in a quiet room and fitted with a Muse headset. They were allowed to place the headset on themselves or receive assistance from the researcher. The headset’s position was adjusted to optimize the EEG signal quality, which was monitored in real time using a custom visualizer in MATLAB R2024a (The MathWorks, Inc., Natick, MA, USA). Word pairs were presented on a Windows 10 laptop running the open-source software PsychoPy 2023.2.2 [5]. The laptop screen was positioned 3 feet from the participant at a visual angle of approximately four degrees. An external monitor connected to the laptop allowed the researcher to monitor the EEG data concurrently. Participants were instructed to judge each word pair’s semantic relatedness and press “R” for related and “U” for unrelated. They first completed eight practice trials. Then, the main task consisted of two blocks of 56 trials, separated by a short break. Each trial began with a prime word displayed for 500 ms, followed by a 500 ms blank screen before the target word presentation. The target word remained on the screen until the participant responded. Two alternative sets of 112 word pairs were created and counterbalanced across participants to ensure that all target words appeared in both the related and unrelated conditions, while preventing any word from being repeated within a participant’s set. The entire experimental session lasted approximately 10 min, including the setup of the Muse headset.

### 2.4. EEG Data Acquisition

**Figure 1:**
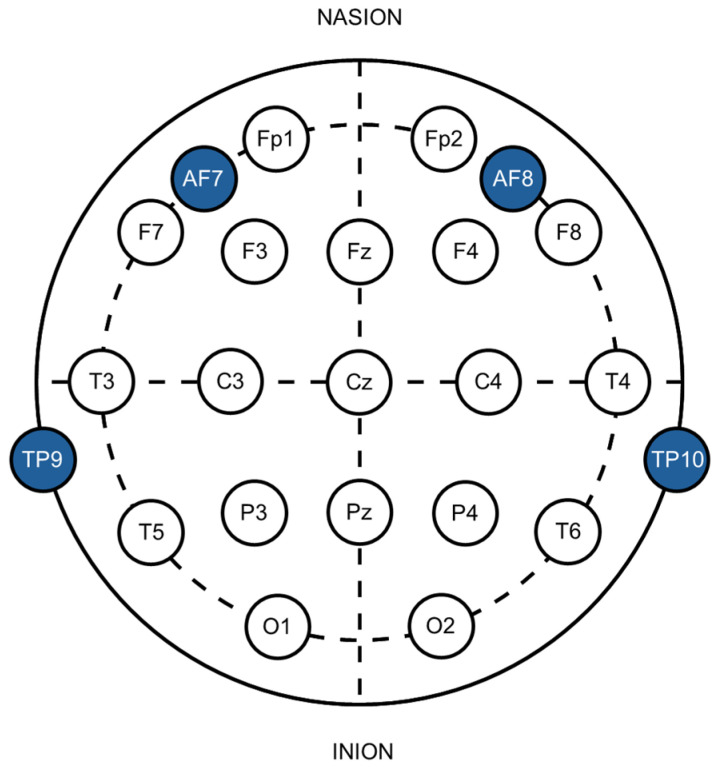
Placement of Muse 2 headset electrodes (dark blue) according to the 10–20 International System (AF = anterior frontal, TP = temporoparietal).

The Muse 2 headband was positioned on the participant’s head, resting across the mid-forehead and fitting over the ears. It includes two sensors located behind the ears (TP9 and TP10), an electrode on each side of the forehead (AF7 and AF8), and a reference electrode situated in the center of the forehead (FPz). These electrode placements correspond to the 10–20 International system [27] commonly used in wired EEG systems (see Figure 1). The open-source BlueMuse software linked the Muse to a laptop via Bluetooth [28] to facilitate connectivity. BlueMuse was also responsible for streaming the raw EEG data using the LSL protocol.

ERP research requires the ability to time-lock precise EEG recordings with stimulus presentation. PsychoPy was selected to present stimuli due to its capacity for streaming LSL timestamp markers and its GUI’s flexibility for incorporating custom Python code. We added code to calculate the exact screen refresh rate, monitor each frame, and send timestamped markers (via LSL) representing the target word onset and condition (Custom codes available on OSF at https://osf.io/u6y9g/, accessed on 11 November 2024). The incoming LSL data streams from BlueMuse and PsychoPy were then saved into a single time-synchronized Extensible Data Format (XDF) file via LabRecorder (stream capture application included in the LSL ecosystem).

### 2.5. EEG Data Processing

Data processing was performed in MATLAB R2024a using the EEGLAB toolbox [29]. The raw EEG signal was high-pass-filtered at 0.1 Hz to minimize slow drift and low-pass-filtered at 30 Hz to reduce excess noise. N400 studies typically use sensors placed on the mastoids (i.e., part of the skull right behind the ears) as references [30]. The data were thus re-referenced offline to the average of the two temporoparietal sensors (TP9 and TP10) due to their proximity to the mastoids. EEG epochs were extracted from −100 ms to 900 ms relative to the word onset to analyze single-trial ERPs elicited by the target words. A baseline correction was performed by averaging data from 100 ms to 0 ms pre-onset and subtracting this average from the rest of the time points. Epochs containing extreme values exceeding ±75 µV were rejected. Data from one participant were excluded from further analyses because no epoch survived the rejection threshold in one condition. In line with standard practice in N400 research, only trials with correct responses were included in the final analyses.

### 2.6. Statistical Analysis

#### 2.6.1. Behavioral Data Analysis

Behavioral responses were analyzed to assess participants’ attention to the stimuli and task performance. Accuracy rates, calculated as the proportion of correct responses from the total number of trials per participant, were analyzed using a generalized linear mixed model (GLMM) with a binomial distribution and a logit link function. This approach is suitable for binary data and allows for accurate modeling of accuracy rates. Response times (calculated for correct responses only) were analyzed using a GLMM with a Gamma distribution and a log link function to account for the typically skewed distribution of reaction times and the potential for multiplicative effects of semantic relatedness on processing speed. For both analyses, the fixed effect was condition (related vs. unrelated), and participants were included as a random effect with a random intercept. Statistical analyses were conducted in Jamovi version 2.5 [31].

#### 2.6.2. ERP Reliability Analysis

To assess the internal consistency of the Muse N400 data, we employed generalizability theory (G theory) to calculate dependability, which serves as a measure of internal consistency analogous to Cronbach’s alpha from Classical Test Theory (CTT). Like Cronbach’s alpha, dependability is a unitless metric, ranging from 0 to +1, with higher values indicating greater consistency. In contrast to CTT, G theory offers a framework for evaluating dependability by accounting for various sources of measurement error [32]. G theory also offers the advantages of accommodating unbalanced data and analyzing nonparallel forms (i.e., those without equal means, variances, and covariances). This acceptance of unbalanced data is particularly beneficial in ERP research, as ERP data are often inherently imbalanced due to the removal of epochs with poor quality during preprocessing [33].

We used the ERP Reliability Analysis (ERA) Toolbox v0.4.5 [33] to compute dependability estimates for each experimental condition in the 250–600 ms range, which encompasses the typical latency of the N400 response. Trial cutoffs (the minimum trials needed to include a participant’s data in dependability analyses) were determined at a reliability threshold of 0.70 based on previously established guidelines [34]. Overall dependability values were then calculated based on these trial cutoffs. Participants with fewer trials than the cutoff for a given condition were excluded from further analysis. To assess the quality of our ERP data, we used the root mean square (RMS) of the standardized measurement error (SME), as recommended by Luck et al. [35]. The SME for each participant was calculated as the standard deviation of the single-trial mean amplitudes divided by the square root of the number of trials. Subsequently, the RMS of these individual SME values was computed across all participants to obtain the RMS-SME, a global measure of data quality. A lower RMS-SME indicates higher data quality, reflecting less variability in the N400 mean amplitude across the sample.

#### 2.6.3. N400 Effect of Semantic Relatedness

We implemented hierarchical linear modeling with the LIMO EEG plug-in for EEGLAB [36]. A general linear model was first implemented to estimate the beta parameter representing the effect of the semantically related and semantically unrelated conditions using ordinary least squares (OLS) regression to account for within-subject variability across single trials. The within-subject model of each single-trial data at each time point and electrode has the general form Y = β0 + β1X1 + β2X2 + ε with Y being the single trial measurement (i.e., voltage amplitude), β0 the intercept, β1 and β2 the beta coefficients to be estimated for each experimental condition, X1 and X2 the coding for each column corresponding to the type of stimulus in the design matrix, and ε the error term. Yuen’s robust paired *t*-tests were computed on the beta estimates at the group level. A non-parametric temporal clustering approach was used to correct for multiple testing, controlling the family-wise error rate at an alpha level of 0.05 [37]. Null distributions were estimated using a permutation procedure with 1000 iterations [37,38] to derive univariate *p*-values and cluster-forming thresholds. Cluster-based inference was implemented using a cluster-sum statistic based on squared t-values [36,37].

## 3. Results

### 3.1. Behavioral Data

Table 2 presents the descriptive statistics for behavioral performance on the semantic relatedness judgment task. Overall, accuracy rates were high in both the related and unrelated word pair conditions (exceeding 97%), suggesting that participants were attentive and engaged and that the task was a valid measure of semantic processing. The GLMM analysis revealed no significant difference in accuracy rates between the related and unrelated word pair conditions, χ^2^ = 1.46, *p* = 0.22, indicating that the two conditions were equivalent in terms of task difficulty.

A separate GLMM analysis conducted on response times (calculated for correct responses only) revealed a significant difference in response times, χ^2^ = 195, *p* < 0.001, with longer response times observed for semantically unrelated word pairs compared to related pairs (see Table 2). This finding aligns with prior literature reporting a facilitating effect of semantic relatedness [39], further supporting the validity of the task developed to test the effectiveness of the Muse headset in measuring the N400.

### 3.2. EEG Data

#### 3.2.1. Internal Consistency

**Figure 2:**
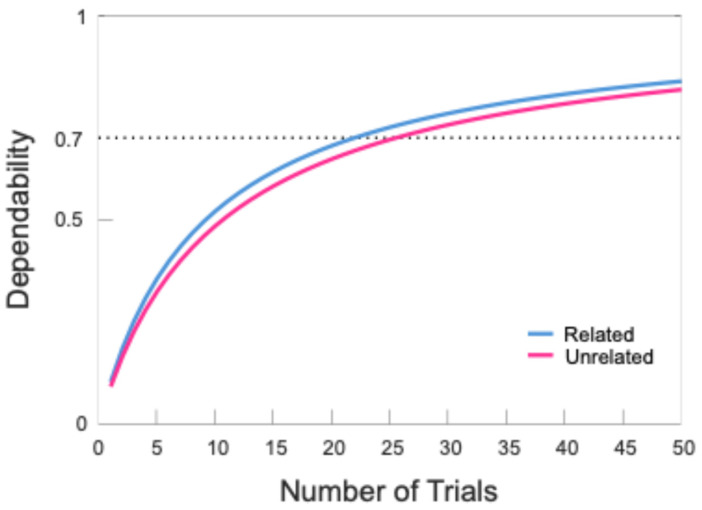
Dependability coefficients of equivalence (internal consistency) for mean N400 amplitudes (250–600 ms) in the semantically unrelated (red trace) and semantically related (blue trace) conditions as a function of the number of trials included. Dependability is a unitless measure of consistency, ranging from 0 to +1. The dotted line represents the reliability threshold which was set at 0.70.

To ensure reliable ERP data, we used the ERA toolbox to determine the number of trials needed to achieve a dependability point estimate of 0.70, a measure of internal consistency analogous to Cronbach’s alpha. Following the recommendations of Clayson and Miller [34], we used trial cutoffs derived from dependability analyses to determine participant inclusion. The results indicated that 24 trials were needed for the semantically related condition and 27 trials for the semantically unrelated condition. The data for all but seven participants met these cutoffs, with an average of 43.17 trials in the semantically related condition and 42.93 trials in the semantically unrelated condition. The overall internal consistency of the included data was high (semantically related = 0.82, semantically unrelated = 0.80). Figure 2 illustrates the relationship between the number of trials and the internal consistency of the N400 time window in both the semantically related and unrelated conditions, and Table 3 shows summary information regarding the number of trials retained for each participant after implementing the cutoffs.

Data quality estimates are presented in Table 4. The total SD (i.e., SD of the mean amplitude in the 250–600 ms time window across all trials and participants) represents the overall variability in the data, including both true signal and noise, while the SME represents the noise component. When the RMS of the SME is smaller than the total SD, it thus suggests that a significant portion of the observed variability in the data is due to actual differences between conditions rather than just measurement noise.

#### 3.2.2. N400 Analysis

**Figure 3:**
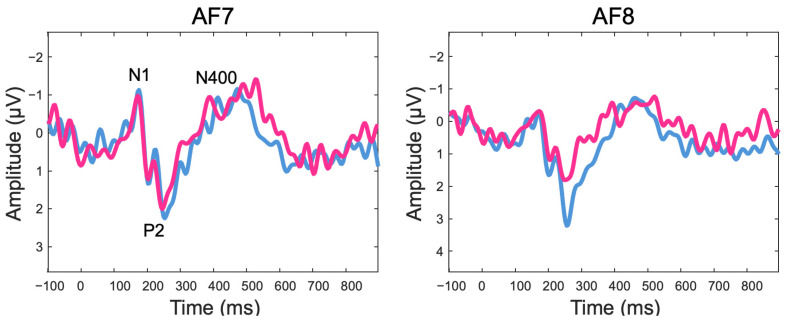
Grand-average waveforms of the ERPs elicited in the semantically unrelated (red trace) and semantically related (blue trace) conditions at the left (AF7) and right (AF8) frontal electrodes.

**Figure 4:**
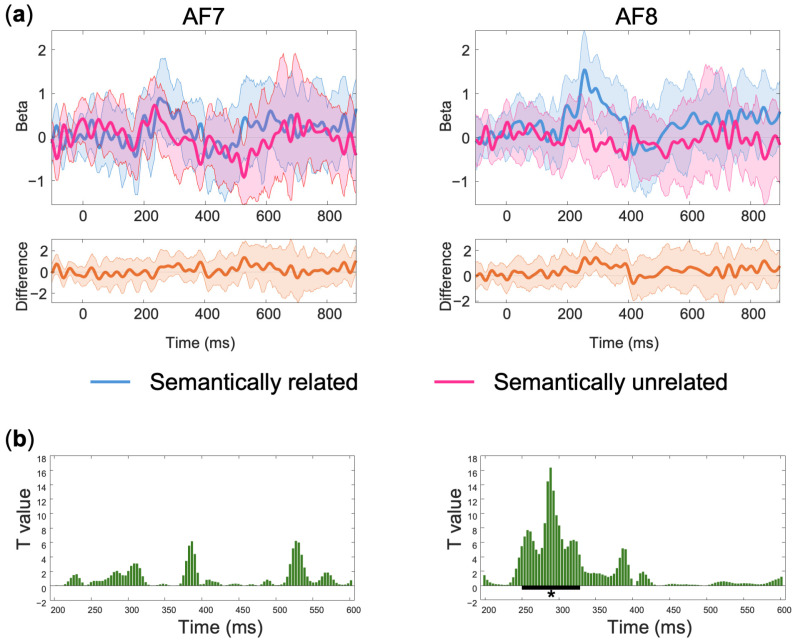
Statistical analysis. (**a**) Modeled amplitudes averaged across subjects for the semantically unrelated (red trace) and semantically related (blue trace) conditions. The shaded area around the beta parameter time courses indicates 95% robust confidence intervals; (**b**) T-values calculated using paired Yuen *t*-tests to compare the two conditions at each individual time point. The thick black line at the bottom of the right plot indicates significant time points after correction for multiple comparisons. The asterisk (*) indicates a significance level of *p* < 0.05.

The grand average of mean subject-level single-trial ERP data is depicted in Figure 3. Several ERP components (N1, P2, N400) can be clearly identified and align well with the existing ERP literature. Additionally, the ERPs time-locked to the onset of the target words showed a more negative deflection in the semantically unrelated condition than the related condition around the N400 time window. This effect was confirmed by the results of the statistical analyses revealing a significant difference over sensor AF8 for a cluster starting at 250 ms and ending at 328 ms (mean difference between semantically unrelated and related conditions = −1.13 microvolts), after correction for multiple comparisons using temporal clustering with a cluster forming threshold of *p* = 0.05 (see Figure 4 for details on the statistical analysis and significance testing).

## 4. Discussion

The present study investigated the efficacy of the Muse 2, a consumer-grade EEG headset, for measuring the N400 component, a language-related ERP associated with semantic processing [17]. Our findings provide compelling evidence that the Muse 2 can reliably capture the N400 effect in a semantic relatedness judgment task, even with its limited electrode configuration. This outcome has important implications for increasing the accessibility and inclusivity of EEG research, particularly in the domain of language and cognition.

### 4.1. Performance of the Muse 2 for N400 Research

Consistent with previous research using traditional EEG systems, we observed a significant N400 effect, with a larger negativity elicited by semantically unrelated word pairs compared to semantically related ones. This effect was evident over the right frontal electrode (AF8), aligning with findings from prior studies showing a slight right-hemisphere bias for the N400 elicited by written words [17]. The successful capture of this effect with the Muse 2 suggests that this affordable and portable device can be a viable tool for investigating semantic processing in language. Note that the high accuracy rates observed in both conditions of the SRJT indicate that participants were attentive and engaged in the task. Moreover, the lack of significant differences in accuracy between conditions confirms that the task difficulty was well balanced, ensuring that the observed N400 effect was driven by semantic relatedness rather than variations in task demands. In conjunction with the ERP findings, the behavioral results thus provide strong support for the suitability of the SRJT paradigm implemented with the Muse 2 headset to measure the semantic N400 effect.

The reliability analysis further strengthens our confidence in the Muse 2’s capability for ERP research. The dependability estimates obtained for the N400 data indicate acceptable internal consistency, suggesting that the Muse 2 can produce reliable ERP waveforms [34] and highlighting its potential suitability for individual differences research. This finding is particularly encouraging given the inherent challenges of using consumer-grade EEG devices for ERP research, such as potential signal quality and noise reduction limitations [3]. However, it is crucial to acknowledge that despite the promising results, consumer-grade devices like the Muse 2 may require more rigorous preprocessing and analysis procedures to account for potential artifacts and lower signal-to-noise ratios than research-grade systems. Furthermore, while Independent Component Analysis (ICA) is a common technique for artifact correction in EEG research [40], its effectiveness on data from the Muse 2 has not yet been systematically established [41].

### 4.2. Expanding Access and Inclusion in EEG Research

The Muse poses solutions to common EEG challenges of equity and inclusion. For instance, a potential benefit is the opportunity to reach previously inaccessible populations. Studies conducted in a university laboratory are often limited to undergraduate students seeking class credit or community members seeking compensation. Restricting studies to these demographics limits the scope of research on literacy, learning disabilities, and development, creating a significant barrier to generalizability. A researcher using the Muse can collect data at a school and reach a desirable sample size of a population that is difficult to reach in a relatively short period of time. In addition to schools, Muse studies can be conducted at locations with greater access to target and marginalized populations such as adult literacy centers and English as a Second Language programs.

A noteworthy advantage of the Muse headset lies in its potential to facilitate the inclusion of diverse participants, addressing the underrepresentation of people of color in ERP research often attributed to barriers associated with hair type and style [42]. Unlike traditional EEG systems that rely on elastic caps, often incompatible with various hairstyles or cultural head coverings, the headband design of the Muse 2, with its placement on the forehead and use of dry sensors, offers greater adaptability. Participants do not have to worry about wetting their hair or removing conductive gel, as these ultra-portable EEG devices are less invasive, more comfortable, and easier to adjust than a traditional EEG net or cap. This allows for the inclusion of participants with a wider range of hair or headwear preferences, including those wearing headscarves, protective styles, or other culturally significant adornments.

The Muse headset and other low-cost mobile EEG devices also offer the opportunity to democratize language ERP research. Researchers without the funding to purchase a traditional EEG system can use the Muse as an effective alternative to wired systems. This can offer early-career researchers in fields with less funding for neuroscience research the ability to conduct language ERP research without being limited by financial and logistical barriers. Similarly, the Muse offers a straightforward alternative for students who lack the experience or the timeframe to conduct EEG studies with a wired system independently.

### 4.3. Limitations and Future Directions

Despite the promising results, it is essential to acknowledge some limitations. First, the Muse 2 utilizes only four electrodes positioned on the frontal and temporal areas of the scalp, compared to the more comprehensive coverage offered by research-grade EEG systems with numerous electrodes placed according to the international 10–20 system. This limited spatial coverage may impact the ability to localize the source of brain activity precisely and could potentially lead to challenges in isolating activity from specific brain regions. However, the Muse headset’s limited electrode configuration may not be a significant barrier in specific research contexts. For instance, when investigating ERP components with well-known and focused scalp distributions, such as the frontal N400 demonstrated in this study or the P300 [13], the Muse 2 can effectively capture these effects. Additionally, if the research question primarily concerns the timing and amplitude of ERP components rather than their precise spatial location, the Muse 2 can provide valuable insights, particularly in studies examining cognitive training or language learning where temporal dynamics are prioritized. It is also worth noting that, especially when the scalp distribution of ERP components of interest does not align with the Muse 2’s electrode locations, other mobile EEG systems may offer complementary electrode configurations [3], potentially addressing some of the limitations of the Muse 2 while retaining the benefits of portability and ease of use.

Additionally, our study focused on the N400 component; further research is needed to assess the Muse 2’s sensitivity to other language-related ERPs, such as the ELAN and P600 related to morpho-syntactic processing [43], as well as its applicability to different experimental paradigms. Indeed, this study focused on a relatively simple semantic judgment task. Future research could explore the use of the Muse 2 in more complex language processing paradigms, such as those involving sentence processing [44], discourse comprehension [45], or bilingual language processing [46].

Furthermore, our sample consisted primarily of university students. Future research should prioritize the inclusion of a more diverse participant pool, encompassing individuals from various age groups, socioeconomic backgrounds, and cultural groups. This broader sampling strategy would not only enhance the generalizability of findings but also provide crucial insights into whether the Muse 2, with its adaptable design, can truly overcome the barriers associated with traditional EEG systems and extend the benefits of accessible neurotechnology to a wider population.

Finally, while the present experiment demonstrated the feasibility of using the Muse headset to examine the effects of semantic relatedness on brain activity, it did not include a direct comparison with research-grade EEG systems. To further establish the validity of the Muse 2 as a tool for measuring N400, future research should directly compare its performance with research-grade EEG systems. Ideally, this comparison would involve a simultaneous recording setup among participants engaged in the same task. Such an approach would facilitate an examination of convergent validity by assessing the correlation between N400 measurements obtained from both devices. Furthermore, it would aid in identifying any systematic differences in the data, thereby highlighting potential limitations of the Muse 2 and contributing to a more comprehensive understanding of its capabilities and validity for N400 research.

## 5. Conclusions

This study demonstrates the potential of the Muse 2 to expand the accessibility and inclusivity of EEG research. Its affordability and portability make it a promising tool for researchers with limited resources, allowing them to conduct EEG studies that might not be feasible with traditional systems. Moreover, the Muse 2’s user-friendly design and ability to be used in real-world settings can facilitate the inclusion of diverse participant populations, including children, older adults, and individuals with mobility impairments, who may face barriers to participating in laboratory-based research. This can potentially enhance the generalizability and ecological validity of EEG research findings. By demonstrating the feasibility of using the Muse 2 to measure the N400 effect, this study opens new avenues for research in language and cognition. Future studies could utilize this approach to investigate individual differences in semantic processing, assess language comprehension in clinical populations, and explore the neural correlates of language learning and development. By making EEG technology more accessible, we can accelerate discoveries in language and cognition and promote a more inclusive and equitable approach to brain research.

## Figures and Tables

**Table 1:** Participant demographics.

		M	SD	Frequency (%)
Age (years)		19.95	3.81	-
Sex (female)		-	-	51.35
Race				
	Asian	-	-	2.70
	Black	-	-	8.11
	Other	-	-	8.11
	White	-	-	81.08
Ethnicity (Hispanic)		-	-	8.33
Handedness (Right)		-	-	86.49
Highest Education				
	High School	-	-	48.65
	Some College	-	-	40.54
	Bachelor’s Degree	-	-	8.11
	Master’s Degree	-	-	2.70

**Table 2:** Descriptive statistics for behavioral measures.

		Condition	
Variable		Related	Unrelated
Accuracy Rate (%)	M	97.06	97.64
	SD	16.91	15.20
Response Time (ms)	M	813.71	984.11
	SD	415.31	514.33

**Table 3:** Overall dependability estimates.

Condition	Threshold	N		Dependability	Trials			
		Included	Excluded		M	SD	Min	Max
Match	0.70	29	7	0.82 CI [0.71 0.90]	43.17	9.04	24	55
Mismatch	0.70	29	7	0.80 CI [0.67 0.89]	42.93	8.29	27	54

**Table 4:** Data quality estimates.

Condition	Total SD	SME		
		RMS	Min	Max
Match	1.46	0.96	0.46	1.93
Mismatch	1.83	1.00	0.41	1.95

## Data Availability

The original data presented in the study are openly available in the Muse Validation for ERP Repository at https://osf.io/u6y9g/ (accessed on 11 November 2024).
